# *Artemisia argyi* Levl.et Vant Extract (AALE) and Parthenolide Suppress Respiratory Syncytial Virus (RSV) via the RIG-I/TLR3 Pathway In Vivo and In Vitro

**DOI:** 10.3390/ph19040640

**Published:** 2026-04-18

**Authors:** Zeting Tan, Rongshun Liang, Adam Junka, Haoxuan Sun, Jie Jiang, Haojia Ma, Shisong Fang, Yanfang Sun

**Affiliations:** 1 College of Life Sciences and Medicine, Zhejiang Sci-Tech University, Hangzhou 310018, China; zeting408506@163.com (Z.T.); rongshun_leong@163.com (R.L.); 2 Zhejiang International Joint Laboratory of Traditional Medicine and Big Health Products Development, Hangzhou 310018, China; 3 “PUMA” Platform for Unique Model Applications, Faculty of Pharmacy, Wroclaw Medical University, Borowska 211, 50-534 Wroctaw, Poland; adam.junka@umw.edu.pl; 4 School of Public Health, Southern Medical University, Guangzhou 510515, China; haoxuansun4@gmail.com; 5 Shenzhen Center for Disease Control and Prevention, Shenzhen 518055, China; mhjpku@foxmail.com (J.J.); szjiangjie@139.com (H.M.)

**Keywords:** respiratory syncytial virus (RSV), *Artemisia argyi* Levl.et Vant extract (AALE), parthenolide, RIG-I/TLR signaling pathway

## Abstract

**Background**: Respiratory syncytial virus (RSV) is a leading global pathogen of acute lower respiratory tract infection, posing significant risks to infants, the elderly, and immunocompromised patients. *Artemisia argyi* Levl.et Vant Extract (AALE) and its active components have a variety of pharmacological effects, but their anti-RSV potential remains unclear. The aim of this study is to investigate the anti-RSV activity of AALE and parthenolide and its underlying mechanisms. **Methods**: Cell counting kit-8 (CCK-8) assay was used to determine the anti-RSV activities of AALE and parthenolide. Time-of-addition assay and phase of action analysis were used to explore the effect of drugs on the viral replication cycle. Quantitative polymerase chain reaction (qRCR), immunofluorescence (IF) and Western blot (WB) were used to investigate the effects of AALE and parthenolide on RSV-F gene and protein and on RIG-I/TLR-3 pathway related molecules in vitro. In vivo antiviral efficacy was verified by hematoxylin–eosin (HE) staining for lung histopathology, quantitative real-time PCR (qPCR) quantification of RSV-F, RIG-I, TLR-3, IRF3, IL-6, and IFN-β gene expression in lung tissues, and enzyme-linked immunosorbent assay (ELISA) for serum IL-6 and IFN-β levels. **Results**: AALE exhibited the strongest anti-RSV activity among the extracts (SI = 27.6), while parthenolide was the most potent monomeric compound (SI = 8.19). In vitro, both AALE and parthenolide were effective in the co-treatment and post-treatment models, reducing RSV-F gene and F protein levels in infected cells. Furthermore, they alleviated RSV infection by regulating RIG-I and TLR-3 pathway-related genes and proteins. In vivo, AALE and parthenolide suppressed lung index and RSV proliferation, attenuated lung injury, and down-regulated RIG-I, TLR-3, IRF3, IL-6, and IFN-β expression in the lungs of RSV-infected mice. **Conclusions**: AALE and its component parthenolide can inhibit the invasion and replication of RSV, making it a potential candidate for the treatment of RSV-related diseases.

## 1. Introduction

Respiratory syncytial virus (RSV) is a single-stranded negative-sense RNA virus belonging to the Paramyxoviridae family and the genus Pneumovirus [1]. It is the main cause of acute lower respiratory tract infections worldwide. It poses a particularly serious threat to vulnerable groups, including infants under two years old, the elderly and individuals with weakened immune systems, leading to symptoms ranging from mild upper respiratory tract inflammation to life-threatening bronchiolitis and pneumonia [2]. Epidemiological data show that by the age of two, RSV infects almost all children worldwide, causing millions of hospitalizations and hundreds of thousands of deaths in children under 5 years old every year [3]. Although it has imposed a huge burden on public health, there is still no effective vaccine that can be widely used at present [4]. The only approved antiviral drug, ribavirin, has limited efficacy and potential teratogenic risks, which limit its clinical application [5]. Therefore, the development of safe and effective anti-RSV drugs with novel mechanisms of action is an urgent medical need.

A large number of studies have shown that the natural products derived from traditional Chinese medicine have low-toxicity antiviral activity [6,7]. As a famous Chinese herbal medicine, *Artemisia* L. species has rich pharmacological value and is widely used to treat various diseases. For example, *Artemisia caruifolia* Buch. Ham. ex Roxb can be used to treat malaria caused by Plasmodium parasites, as well as to treat a variety of symptoms caused by viral and bacterial infections and reduce inflammatory responses [8,9]. *Artemisia argyi* Levl.et Vant (*A. argyi*) is a perennial herbaceous plant in the family Asteraceae, distinguished by its strong aromatic odor, erect and robust stem, and thick, papery leaves densely covered with grayish-white pubescence on both surfaces [10]. Phytochemical analyses have identified a rich profile of bioactive constituents in the *A. argyi* Levl.et Vant extract (AALE), including flavonoids, terpenoids (e.g., monoterpenes, sesquiterpenes, and triterpenes), phenylpropanoids, and organic acids [11]. Contemporary pharmacological research has demonstrated that AALE possesses a broad spectrum of biological activities, most notably potent anti-inflammatory, antioxidant, antimicrobial, antiviral, and immunomodulatory effects [12]. Previous studies have reported that *A. argyi* can inhibit the replication of influenza viruses and coronaviruses by regulating the host immune response and blocking viral entry. However, its anti-RSV activity and mechanism remain unclear, hindering its development as a potential anti-RSV agent [13,14]. In our previous study, 100 chemical constituents in AALE were identified by UHPLC-MS/MS, including 19 flavonoids, 6 terpenoids, 10 organic compounds and 5 amino acids. A total of 74 constituents were identified in *Artemisia caruifolia* Buch-Ham extract (ACBE), including 10 flavonoids, 12 organic compounds, 5 alkaloids and 6 terpenes. A total of 65 constituents were identified in *Artemisia capillaris* Thunb extract (ACTE), including 11 kinds of flavonoids, 10 kinds of organic compounds, 4 kinds of organic bases, and 3 kinds of alkaloids [15]. Flavonoids and terpenoids accounted for a high proportion in *Artemisia* extract, and parthenolide was one of the terpenoids, which was the common key active ingredient in the extract.

Parthenolide, a sesquiterpene lactone compound, was first isolated from the aerial part of *Tanacetum parthenium* and is also present in AALE [16], and it is another natural product with multiple biological activities such as anti-inflammation, anti-cancer and anti-virus [17]. Recent studies have shown that parthenolide can inhibit the replication of various viruses, including herpes simplex virus type 1 and SARS-CoV-2, and its antiviral mechanism is mainly associated with the ability to regulate host cell signaling pathways that are crucial for viral replication and the host immune response [18,19,20]. However, whether parthenolide has anti-RSV activity and its specific molecular mechanism have yet to be reported.

Given the limitations of current anti-RSV treatment methods and the potential antiviral properties of AALE and parthenolide, this study aims to systematically investigate the anti-RSV effects of AALE and parthenolide in vitro and in vivo. Specifically, the component with higher activity, namely parthenolide, was first screened from seven monomer compounds. Then, the effects of parthenolide and AALE on RSV replication were detected at the cell level, and the mechanism by which they exert antiviral activity by regulating the RIG-I/TLR3 signaling pathway was clarified. Finally, we verified the efficacy and safety of AALE and parthenolide against RSV in an RSV-infected mouse model. This study provides experimental evidence supporting the development of AALE and parthenolide as novel anti-RSV therapeutic agents. Additionally, our findings deepen the understanding of the molecular mechanisms underlying the resistance of natural products to RSV infection.

## 2. Results

### 2.1. In Vitro Experimental Results

#### 2.1.1. 50% Tissue Culture Infective Dose (TCID_50_) Assay

To determine the titer of RSV, the cells with lesions were observed and marked under a microscope. The cytopathic effect (CPE) of different dilutions of RSV is shown in Table 1. The TCID_50_ of RSV was calculated as 10–5.64/mL using the Reed–Munch method. Subsequently, the experiment was conducted with a virus at an MOI = 0.1.

#### 2.1.2. Anti-RSV Activity of AALE and Parthenolide In Vitro

CCK-8 assay was used to detect the cytotoxicity and antiviral activity of *Artemisia* extract and its monomer compounds on Hep-2 cells. Ribavirin was used as the positive control. The IC_50_ (50% Inhibitory Concentration), EC_50_ (50% Effective Concentration) and SI values of the extracts and compounds are shown in Table 2, respectively. In antiviral research on natural products, SI ≥ 10 is usually considered as a reference threshold for further research of plant extracts or pure compounds, while SI > 20 is considered as an important index with further development potential [21,22,23]. As shown in Table 2, the selective index (SI) of the extracts was all above 20. Among them, the extract from *A. argyi* had the highest SI value (SI = 27.62), indicating that its antiviral effect was the best. Among the seven tested monomeric compounds, parthenolide exhibited the highest selectivity index (SI =8.19), which was comparable to the reference standard of pure compounds, suggesting that parthenolide possesses anti-RSV activity. Dihydroartemisinin (SI = 2.14), artemether (SI = 2.95), and costunolide (SI = 3.4) also had certain inhibitory effects on RSV, but the effects were not significant. Artesunate, atractylenolide, and dehydrocostuslactone had no obvious therapeutic effect on RSV. Subsequently, further research was conducted using the AALE and parthenolide.

**Figure 1:**
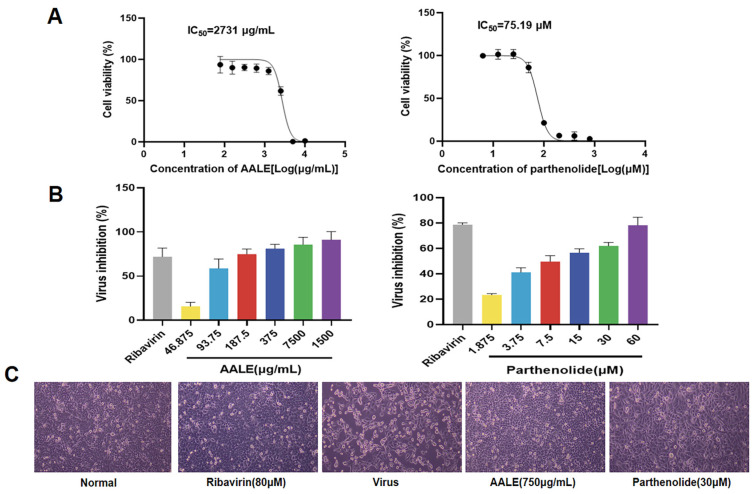
Anti-RSV activity of AALE and parthenolide. (**A**) Cytotoxicity assay of AALE and parthenolide on Hep-2 cells. (**B**) Inhibition rate of RSV after treatment with different concentrations of AALE or parthenolide. (**C**) Effect of AALE (750 μg/mL) or parthenolide (30 μM) on the state of RSV-infected Hep-2 cells under the microscope (original magnification: 100×).

The cytotoxic effects of AALE and parthenolide on Hep-2 cells are shown in Figure 1A, and the anti-RSV activity is shown in Figure 1B. AALE and parthenolide inhibited viral infection in a concentration-dependent manner within the safe concentration range. After 48 h of RSV infection, the morphology of cells in different treatment groups was observed by microscope. According to the results, compared with the virus control group, the cells treated with AALE or parthenolide were not significantly different from the normal control group and the ribavirin group, and still remained in good condition (Figure 1C). Collectively, these data suggest that AALE and parthenolide exhibit significant inhibitory effects against RSV.

#### 2.1.3. Identification of the Effective Stage of AALE and Parthenolide Against RSV Infection

**Figure 2:**
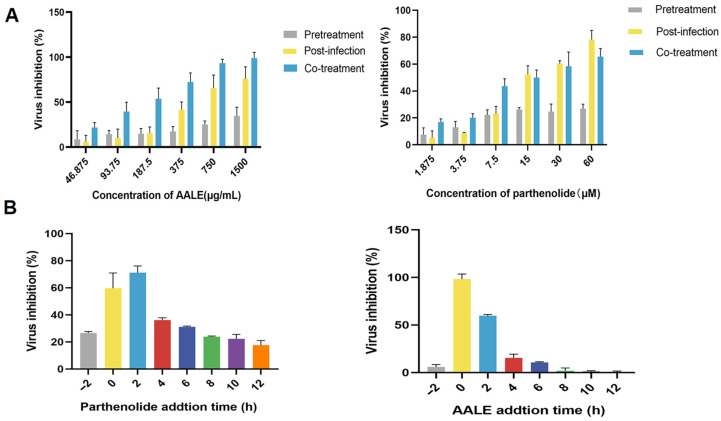
Antiviral effects of AALE and parthenolide on RSV at different treatment phases and timing of administration. (**A**) Virus inhibition rate of AALE and parthenolide in three treatment modes (Pretreatment: Pre-incubation with drug before RSV infection; Post-infection: Drug addition after RSV adsorption; Co-treatment: Simultaneous incubation of drug and RSV). (**B**) Virus inhibition rate of AALE and parthenolide when administered at different time points relative to RSV infection.

In order to explore the mechanism and effective action stage of the AALE and parthenolide on RSV, three different action stages were designed, namely pre-treatment (viral adsorption inhibition), post-infection (viral proliferation blocking), and co-treatment (immune protection activation). As shown in Figure 2A, AALE showed the highest viral inhibition rate in the co-treatment stage, followed by the post-infection stage, while parthenolide showed the highest viral inhibition rate in the post-infection stage, followed by the co-treatment stage. In the pre-treatment stage of the virus, both showed almost no inhibition. The results showed that the antiviral effect of AALE and parthenolide mainly occurred during and after the entry of RSV into the cells and had the effects of blocking virus invasion, activating cell immunity and inhibiting virus proliferation.

#### 2.1.4. Effects of Different Administration Times on RSV

Viral replication is mainly involved in virus adsorption, penetration, biosynthesis, assembly and release. Either 1500 μg/mL of AALE or 60 μM of parthenolide was added at eight time points before and after RSV infection of Hep-2 cells to determine the effect of AALE and parthenolide on the viral replication cycle. The results showed that AALE had the highest inhibition rate of virus at 0 h (added simultaneously with virus), followed by 2 h, while parthenolide had the highest inhibition rate of virus at 2 h after virus infection, followed by 0 h. With the extension of treatment time, the inhibition effect of both on virus replication was gradually weakened. However, AALE exerted little to no inhibitory effect on viral replication at the 6 h post-infection time point (Figure 2B). This suggests that AALE and parthenolide mainly inhibit virus invasion and proliferation in host cells.

#### 2.1.5. AALE and Parthenolide Inhibit RSV Replication in the Co-Treatment Mode

**Figure 3:**
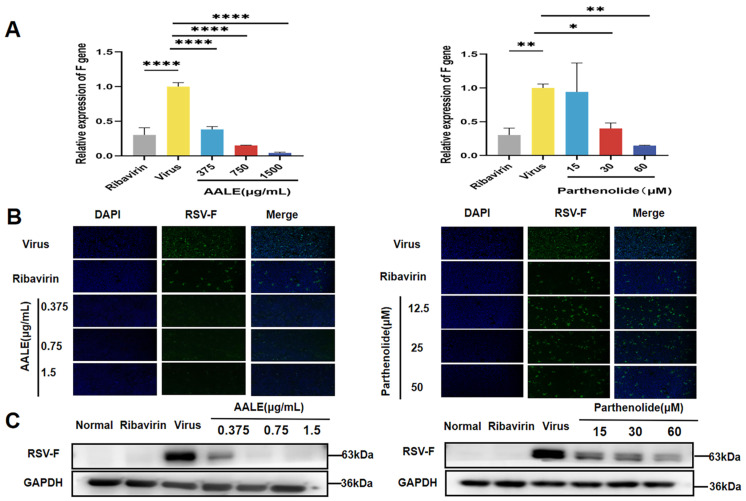
AALE and parthenolide inhibited RSV replication by reducing RSV-F gene and protein expression in the co-treatment mode. (**A**) Relative expression of RSV-F gene mRNA in cells after co-treatment with AALE or parthenolide and virus. (**B**) Immunofluorescence analysis of RSV-F protein after co-treatment of cells with AALE or parthenolide and virus (original magnification: 40×). (**C**) Western blot analysis of RSV-F protein levels after co-treatment of cells with AALE or parthenolide and virus. * *p* < 0.05, ** *p* < 0.01, **** *p* < 0.0001 was compared with virus group.

The RSV-F protein, as the key envelope glycoprotein of RSV, mainly functions to mediate the fusion of the viral envelope and the host cell membrane, providing necessary conditions for the entry of the viral genome into the host cells, and is a key step in initiating the RSV replication cycle. To further determine the inhibitory effect of AALE and parthenolide on viral replication in RSV-infected Hep-2 cells, RSV-F gene expression and F protein levels were measured in cells incubated with virus and test compounds simultaneously in the co-treatment mode. As shown in Figure 3A, AALE treatment significantly reduced F gene expression in a dose-dependent manner compared to the viral control, as did the positive control ribavirin. Immunofluorescence assay showed that F protein (green fluorescence) was significantly reduced in the cells treated with AALE or parthenolide, while the virus control group showed a strong fluorescence signal (Figure 3B). Western blot analysis showed that AALE and parthenolide inhibited the expression of F protein in the co-treatment mode compared with the viral control (Figure 3C). These results suggested that in the combined treatment mode, AALE or parthenolide might competitively block the adsorption and invasion of the virus to host cells by directly acting on the virus particles or blocking the key adsorption proteins on the surface of the virus. In addition, they may also inhibit viral replication and proliferation by inhibiting key steps such as viral genome transcription and protein synthesis.

#### 2.1.6. AALE and Parthenolide Inhibit RSV Replication in the Post-Infection Mode

**Figure 4:**
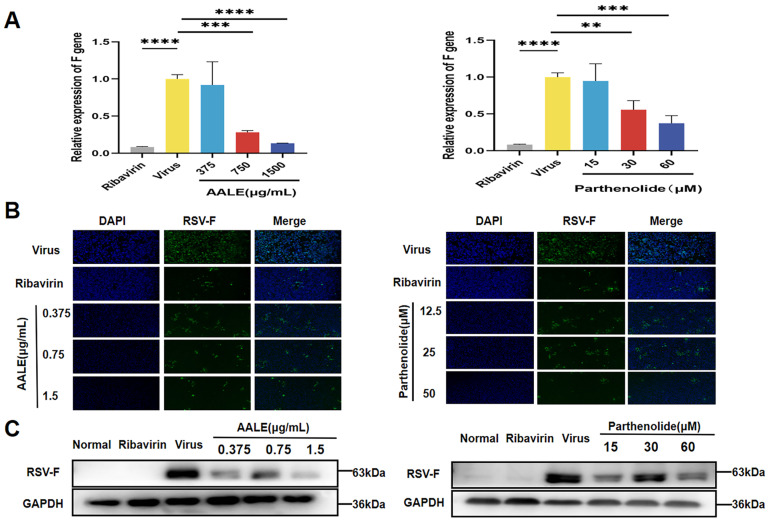
AALE and parthenolide inhibited RSV replication by reducing RSV-F gene and protein expression in post-infection mode. (**A**) Relative mRNA expression of RSV-F gene in virus-infected cells treated with AALE or parthenolide. (**B**) Immunofluorescence analysis of RSV-F protein in virus-infected cells treated with AALE or parthenolide (original magnification: 100×). (**C**) Western blot analysis of RSV-F protein levels in virus-infected cells treated with AALE or parthenolide. ** *p* < 0.01, *** *p* < 0.001, **** *p* < 0.0001 was compared with virus group.

Similarly, we assessed intracellular viral F gene expression and F protein levels in Hep-2 cells in a post-infection treatment mode. As shown in Figure 4A, treatment with AALE or parthenolide significantly reduced the expression of the RSV-F gene in cells compared with the viral control group, showing the most significant inhibition at the highest concentration. Consistent with the gene expression results, cellular immunofluorescence and Western blot analyses showed that treatment with AALE or parthenolide significantly inhibited RSV-F protein expression (Figure 4B,C). These results suggested that AALE and parthenolide inhibited RSV proliferation in the dormitory cells.

#### 2.1.7. Effect of AALE and Parthenolide on the Expression of Cytokines Involved in RIG-I and TLR-3 Signaling Pathways

RIG-I and TLR-3 are signal transduction pattern recognition receptors. They play a crucial role in the innate immune response to viral infections. However, excessive activation of this natural immune signal is harmful as it leads to the overexpression of pro-inflammatory cytokines, interferons, and other downstream mediators, causing severe damage to the organism. Total RNA and protein were extracted from the cells to detect the expression of related cytokines and proteins in different treatment groups.

**Figure 5:**
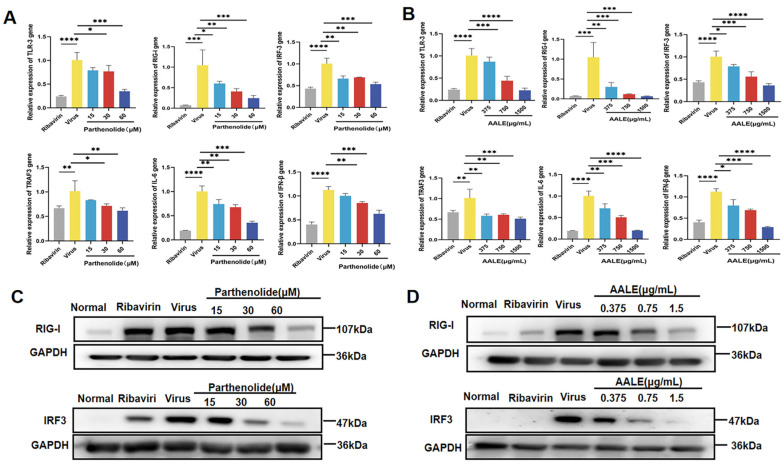
Regulation of TLR3 and RIG-I signaling pathways by parthenolide or AALE in RSV-infected Hep-2 cells. (**A**,**B**) qRT-PCR analysis of relative mRNA expression of key genes in the TLR3 and RIG-I signaling cascades, including TLR3, RIG-I, IRF3, TRAF3, IL-6, and IFN-β, in virus-infected cells following treatment with parthenolide (**A**) or AALE (**B**). (**C**,**D**) Western blot analysis of RIG-I and IRF3 protein expression in virus-infected cells treated with parthenolide (**C**) or AALE (**D**). * *p* < 0.05, ** *p* < 0.01, *** *p* < 0.001, **** *p* < 0.0001 was compared with virus group.

As shown in Figure 5A,B, the expression levels of RIG-I, TLR3, IRF3, TRAF3, INF-β, and IL-6 genes were significantly increased after RSV infection. After the 48 h treatment of cells with AALE or parthenolide, the relative expression of each of these related genes was significantly down-regulated in a concentration-dependent manner compared with the viral control. Western blot analysis showed that RSV infection increased the levels of RIG-I and IRF3 phosphorylated proteins in Hep-2 cells, and AALE or parthenolide treatment significantly decreased the levels of RIG-I and IRF3 phosphorylated proteins compared with the virus control group (Figure 5C,D). These results suggest that AALE and parthenolide exert their antiviral effects by regulating RIG-I and TLR3 signaling pathways.

### 2.2. In Vivo Experimental Results

#### 2.2.1. Protective Effect of AALE and Parthenolide on Lung Tissue Injury Induced by RSV in Mice

**Figure 6:**
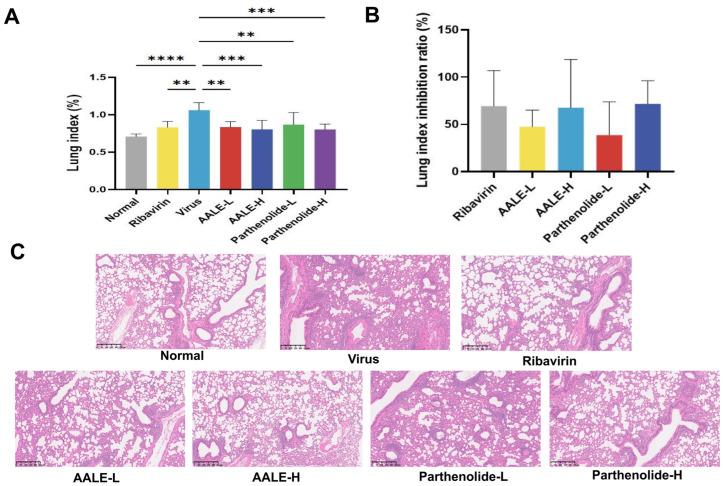
AALE and parthenolide alleviate RSV-induced lung injury in mice. (**A**) Lung index of mice in different treatment groups (*n* = 6). (**B**) Lung index inhibition rate of each treatment group. (**C**) Hematoxylin–eosin (HE)-stained lung tissue sections (100×). Normal: uninfected control; Virus: RSV-infected control; Ribavirin: positive control; AALE-L/AALE-H: low/high dose of AALE; Parthenolide-L/Parthenolide-H: low/high dose of parthenolide. ** *p* < 0.01, *** *p* < 0.001, **** *p* < 0.0001 was compared with virus group.

The lung index and lung index inhibition rate can be used to reflect the severity of lung injury [24]. The higher the lung index, the more serious the lung tissue lesions, and the higher the lung index inhibition rate, the better the drug treatment effect. After mice were infected with RSV, the lung index significantly increased (Figure 6A). This is because the infection of RSV in mice causes inflammatory infiltration, pulmonary congestion, thickening of the alveolar walls, and severe interstitial congestion, thereby increasing the lung weight. After treatment with ribavirin, AALE or parthenolide, the lung index of mice was significantly lower compared to the virus control group, and the lung index inhibition rate increased (Figure 6B). The HE staining results showed that compared to the normal control group, the lung tissue of mice in the virus control group had inflammatory cell infiltration. Compared to the virus control group, the inflammatory infiltration and edema in the lung tissue of mice in the high-dose AALE and parthenolide groups were significantly reduced (Figure 6C), and the lung tissue of mice in the low-dose group had a certain degree of lesion. The above results show that AALE and parthenolide can alleviate lung injury caused by RSV infection.

#### 2.2.2. AALE and Parthenolide Reduce Pulmonary Viral Load in RSV-Infected Mice

**Figure 7:**
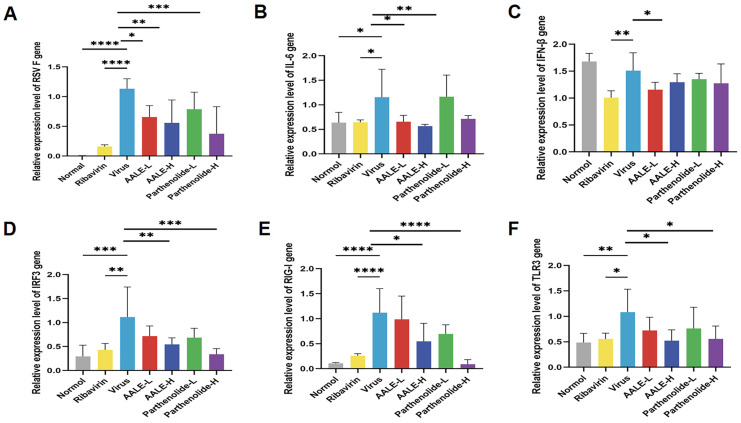
AALE and parthenolide modulate RSV load and the expression of RIG-I/TLR3 signaling pathway-related genes in lung tissues of RSV-infected mice (*n* = 6). (**A**) Relative RSV-F gene expression in mouse lung tissue. (**B**–**F**) Relative gene expression of IL-6, IFN-β, IRF3, RIG-I, and TLR3 in mouse lung tissues. * *p* < 0.05, ** *p* < 0.01, *** *p* < 0.001, **** *p* < 0.0001 was compared with virus group.

The viral load of RSV (RSV) in the lung tissue of mice is a direct indicator for directly measuring the viral replication level of RSV in the mice’s bodies and evaluating the antiviral efficacy of related drugs [25]. By detecting the expression level of the RSV-F gene in lung tissue, the replication degree of RSV in mice was determined. The results showed that the expression of the RSV-F gene in the virus infection group was significantly higher than that in the blank control group. After treatment with AALE or parthenolide, RSV-F gene expression was inhibited in a dose-dependent manner, and the inhibition effect of AALE on RSV-F gene expression was better than that of parthenolide (Figure 7A).

#### 2.2.3. Relative Expression of RIG-I, TLR3, IRF3, IFN-β and IL-6 Genes in Lung Tissue of Mice

RIG-I and TLR3 are key pattern recognition receptors for the host recognition of viral RNA, and both of them can phosphorylate and activate IRF3 through downstream signaling axes after activation [26]. The activated IRF3 is transferred to the nucleus to initiate IFN-β transcription and mediate the body’s broad-spectrum antiviral innate immune response [27]. At the same time, this pathway can also promote the release of proinflammatory factors such as IL-6 and participate in antiviral inflammatory response and immune regulation. The effects of AALE and parthenolide on RSV-infected mice were verified by detecting the expression of cytokines related to RIG-I and TLR-3 signaling pathways (RIG-I, TLR-3, IRF3, IFN-β and IL-6) in lung tissues. The results showed that the expression of RIG-I, TLR-3, IRF3, and IL-6 in the lung tissues of mice treated with AALE or parthenolide was significantly higher than that in the virus model group, while the expression of IFN-β was significantly reduced only in the high concentration of AALE group (Figure 7B–F). These results suggested that AALE and parthenolide could exert anti-RSV activity by regulating the RIG-I and TLR-3 signaling pathways and reducing the levels of inflammatory response-related factors.

#### 2.2.4. Effects of AALE and Parthenolide on Serum Levels of IL-6 and IFN-β in RSV-Infected Mice

**Figure 8:**
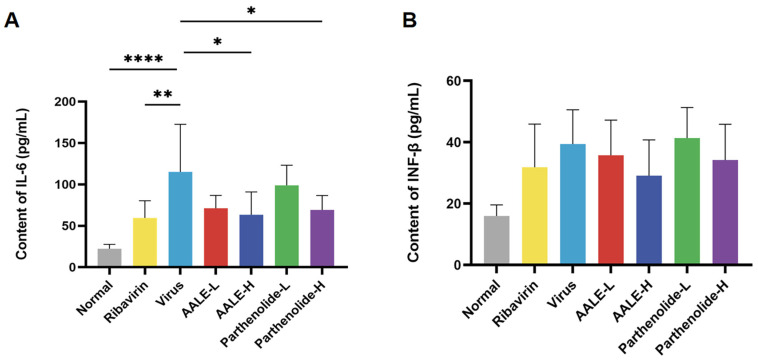
AALE and parthenolide reduced the levels of proinflammatory cytokines in the serum of RSV-infected mice (*n* = 6). Content of IL-6 (**A**) and content of IFN-β (**B**) in mouse serum detected by ELISA. * *p* < 0.05, ** *p* < 0.01, **** *p* < 0.0001 was compared with virus group.

It is known that IFN-β and IL-6 are cytokines related to the RIG-I and TLR3 signaling pathways, and their excessive expression can cause serious damage to the body. By detecting the levels of IFN-β and IL-6 in the serum of mice, we further explored the mechanism of their antiviral effects. After treatment with AALE or parthenolide, IL-6 levels were significantly decreased in the AALE and parthenolide high-dose mice compared with the viral group (Figure 8A), and IFN-β levels also tended to decrease but not significantly in the high-dose group (Figure 8B). In conclusion, AALE or parthenolide treatment inhibited the upstream RIG-I and TLR3 receptors, regulated the levels of downstream cytokines IL-6 and IFN-β, and attenuated the immune–inflammatory response induced by RSV infection in mice.

## 3. Discussion

RSV is an important pathogen that causes severe lower respiratory tract infections in immunocompromised people worldwide. It can cause severe pneumonia and bronchitis, resulting in a large number of hospitalizations and deaths every year. At present, the commonly used broad-spectrum antiviral drug is ribavirin, but it is largely limited by its high price, and more so, its side effects. Therefore, it is imperative to develop alternative drugs for the treatment of safe and effective anti-RSV infections.

*Artemisia* species are found worldwide and consist of about 500 species and subspecies. Some species of this genus, including *A. argyi*, *Artemisia annua* and *Artemisia capillaris*, are used as well-known traditional Chinese medicines for the treatment of a variety of diseases such as malaria, hepatitis and fungal, bacterial, and viral infections, but the chemical components that play the major roles are not clear. *Artemisia* species are rich in a variety of chemical constituents. In our previously published study, 100 chemical constituents in AALE, 74 in ACBE and 65 in ACTE were identified by UHPLC-MS/MS, among which flavonoids and terpenoids accounted for the highest proportion [15]. The three *Artemisia* extracts and their important chemical constituents, including artemisinin derivatives (dihydroartemisinin, artesunate and artemether), parthenolide, atractylenolide, costunolide and dehydrocostus olide, were screened for their anti-RSV activity. The screening results showed that all three *Artemisia* extracts had good anti-RSV activity, and AALE had the best therapeutic effect. Parthenolide, as one of the common components in the extracts, was shown to be the key active component against RSV. Although parthenolide has been identified as a terpenoid in parthenolide, its exact amount in the extract remains unknown. In the future, we could determine the amount of parthenolide in AALE by HPLC/MS quantitative analysis, which would further clarify its contribution to the overall anti-RSV activity of the crude extract.

The normal viral replication cycle is divided into six phases: adsorption, invasion, uncoating, biosynthesis, assembly, and release [28]. From the analysis of the effective action stage and the results of the time addition experiment, it can be seen that AALE and parthenolide mainly play a role in the invasion and replication assembly stages during the viral replication cycle.

RSV-F protein is a trimeric transmembrane glycoprotein that mediates the binding of RSV to cellular receptors and induces fusion between the viral envelope and the cell membrane, which plays an important role in inducing host cellular immune protection [29]. Both AALE and parthenolide significantly reduced the contents of the F gene and the F protein in the cells during the co-treatment stage and the post-treatment stage, which indicated that RSV replication was inhibited to different degrees.

Nonspecific innate immunity is the first line of defense against viral infection. Retinoic acid-inducible gene I-like receptors (RLRs) and TLRs (Toll-like receptors) play an important role in antiviral immunity. RLRs and TLRs are the most important molecular families of intracellular immune recognition of a broad spectrum of microbial patterns, including RIG-I. RIG-I binds to downstream mitochondrial antiviral signaling proteins to stimulate the activation of downstream factor TRAF3, which in turn activates the TBK-1 complex and induces the phosphorylation of IRF-3. The activated IRF3 is transported into the nucleus to induce IFN-β expression, and the activated NF-κB promotes the secretion of IL-6 and other inflammatory factors [30]. Toll-like receptors (TLRs) are type I integral membrane receptors, among which TLR-3 recognizes viral dsRNA and activates IRF3 and NF-κB through TRIF-dependent signaling [31]. The former also promotes the synthesis of IFN-β, and the latter induces the production of pro-inflammatory cytokines such as IL-6 to aggravate the inflammatory response. The key signaling molecules RIG-I and IRF3 were up-regulated in Hep-2 cells after RSV infection. This eventually leads to the high expression of interferon beta and inflammatory factor IL-6. In the co-treatment model, AALE and parthenolide exerted antiviral activities through down-regulating the expression of key proteins RIG-I and IRF3 and down-regulating the expression of RIG-I, TLR3, IRF3, TRAF3, IFN-β, and IL-6 at the gene level. These results suggest that AALE or parthenolide may exert their anti-RSV activities by regulating innate immune response and reducing inflammatory response.

BALB/c mice have become the most widely used experimental animals in the study of RSV infection because of their clear genetic background, convenient experimental operation and relatively low cost. In this study, BALB/c mice were used to establish an RSV infection model, and the anti-RSV effects of AALE and its active component, parthenolide, were evaluated in vivo by detecting lung index, inhibition rate of lung index, lung pathology, viral load, IFN-β and IL-6 levels, and gene expression of RIG-I/TR3 signaling pathway-related factors. According to the overall experimental results, both AALE and parthenolide have certain anti-RSV activity, and the better therapeutic effect of AALE over parthenolide (consistent with the in vitro results) may be due to the fact that the synergistic effect of multiple components and multiple targets in AALE is superior to the effect of a single component. RSV infection can cause inflammatory cell infiltration, hyperemia, and edema in lung tissue [32]. These pathological changes can directly lead to a significant increase in lung wet weight, and body weight may decrease due to reduced food intake after infection. AALE and parthenolide reduced the increase in lung index caused by RSV, and the inhibition rate of lung index increased at the same time. The pathological analysis of lung tissue showed that the degree of lesion was reduced in AALE-H, while AALE-L and parthenolide-L had varying degrees of lesion. The results indicate that AALE is more effective than parthenolide. In addition, the expression of the RSV-F gene in mouse lung tissues was measured by qPCR, and the results showed that both AALE and parthenolide significantly reduced RSV-F mRNA expression and inhibited RSV replication in vivo.

## 4. Materials and Methods

### 4.1. Materials and Reagents

Ribavirin (CAS: 36791-04-5), artemether (CAS: 71963-77-4), dihydroartemisinin (CAS: 71939-50-9), and artesunate (CAS: 88495-63-0) were purchased from Aladdin Biotechnology Co., Ltd. (Xi’an, China). Parthenolide (CAS: 20554-84-1), costunolide (CAS: 553-21-9), atractylenolide (CAS: 73069-13-3), and dehydrocostuslactone (CAS: 477-43-0) were purchased from Puxitang Biotechnology Co., Ltd. (Beijing, China). Cell culture medium (DMEM), fetal bovine serum (FBS), and 0.25% EDTA-trypsin (25200-056), 1% penicillin–streptomycin solution were purchased from Gibco (Waltham, MA, USA). Phosphate-buffered saline (10 × PBS) was purchased from Regen Biotechnology (Seoul, Republic of Korea). RIPA lysis buffer and CCK8 (C0038) were purchased from Biotime (Xiamen, China). FastPure Cell/Tissue Total RNA Isolation Kit (RC101-01) was purchased from Vazyme (Nanjing, China). One Step TB Green^®^ PrimeScript™ RT-PCR Kit II (RR086A) was obtained from Takara (Shiga, Japan). The enzyme-linked immunosorbent assay kit (E-EL-M0044) was purchased from Elabscience (Wuhan, China). Anti-RSV F monoclonal antibody, GAPDH, RIG-I, IRF3, Goat anti-mouse IgG and Goat anti-rabbit IgG were provided by Abcam (Cambridge, UK).

The *A. argyi* were purchased from the Zhejiang Huitong Agricultural Science and Technology Co., Ltd. (Hangzhou, China); the *Artemisia caruifolia* was collected from the Wuling Mountain area of Chongqing, China; and the *Artemisia capillaris* was obtained from the Yimeng Mountain area of Shandong, China. Firstly, supercritical CO_2_ extraction was employed, followed by ultrasonic extraction with ethyl acetate to obtain the extract of the *Artemisia* genus for the subsequent experiments. The extract was dissolved in DMEM, then filtered and sterilized using a 0.22 μM filter.

### 4.2. In Vitro Experiments

#### 4.2.1. Cells and Virus Culture

Human laryngeal carcinoma epithelial cells (Hep-2, No.: 4201PAT-CCTCC00214) and the RSV strain were obtained from the Shenzhen Center for Disease Control and Prevention (Shenzhen, China). The DMEM solution containing 10% fetal bovine serum and 1% penicillin–streptomycin was used as the culture medium for Hep-2 cells, which were cultured in an incubator containing 5% CO_2_ at 37 °C. When Hep-2 cell growth reached 80% confluence, DMEM containing 2% fetal bovine serum was added after washing with PBS three times, and then RSV was inoculated into the cells. When the cytopathic effect (CPE) reached 80%, the virus was harvested, aliquoted, and stored at −80 °C for later use [24].

#### 4.2.2. Determination of Median Tissue Culture Infectious Dose (TCID_50_)

The amplified virus stock solution was serially diluted 10-fold to obtain 8 concentration gradients ranging from 10^−1^ to 10^−8^. Each gradient diluted virus suspension was added to a 96-well plate at a volume of 100 μL per well, with 6 replicate wells. At the same time, a normal cell control group was set up. The cells were cultured for 72 h. During this period, the cell status was observed at any time, and the number of cell Wells with lesions was recorded. The TCID_50_ was calculated by the Reed–Muench method [33].

#### 4.2.3. The Cytotoxicity Experiment of AALE and Parthenolide Hep-2 Cells

Hep-2 cells in the logarithmic growth phase were seeded in 96-well plates at a density of 8000 cells per well. After the cells adhered, a series of different concentrations of *Artemisia* plant extract and monomer compounds were added to the cells, and they were incubated at 37 °C and 5% CO_2_ for 48 h. After incubation, 100 μL of 10% CCK-8 solution was added to each well under light-protected conditions and incubated in the dark at 37 °C for 30 min. The optical density (OD) was detected with a microplate reader at a wavelength of 450 nm, and the cell survival rate was calculated [34].

#### 4.2.4. Screening of Artemisia Extract and Its Active Components Against RSV

Ribavirin was used as the positive control drug. The positive drug and the experimental drug were diluted into a series of different concentrations by the 2-fold dilution method using DMEM containing 2% FBS. The 96-well culture plate covered with a single layer of cells was prepared. Then, 50 μL of RSV disease solution (MOI = 0.1) and 50 μL of different concentrations of drug-containing culture medium were added to each well simultaneously. Three duplicate wells for each concentration of the drug were set up. At the same time, the virus control group and the normal cell control group were established. After treatment for 48 h, the OD values of each well were detected [35] and the virus inhibition rate and selection index (SI) value were calculated. The antiviral effects of each drug were evaluated based on the SI value.

Virus suppression rate (%) = (OD value of the experimental group − OD value of the virus group)/(OD value of the normal group − OD value of the virus group) × 100%; SI = IC_50_/EC_50_.

#### 4.2.5. Analysis of the Effective Anti-RSV Action Stages

To investigate the antiviral activities of AALE and parthenolide at distinct stages of viral replication, three experimental groups were established [36]. Hep-2 cells were first seeded into 96-well plates and cultured at 37 °C in a 5% CO_2_ atmosphere until reaching 90% confluence. Subsequent treatments were performed as follows:

(a) Pretreatment (virus adsorption inhibition): Cells were pretreated with a drug solution at 37 °C and 5% CO_2_ for 2 h. After the solution was discarded, the cells were infected with the RSV solution for 2 h. After discarding the virus solution, the cell surface was washed twice with PBS to remove residual virus. Fresh virus maintenance medium was then added, and the sample was incubated for 48 h.

(b) Co-treatment (immunoprotective activation): Different concentrations of drug-containing culture medium and virus solution were added to the cells together, followed by continued incubation for 48 h.

(c) Post-infection (virus proliferation block): Cells were infected with RSV solution for 2 h. After viral solution removal, cells were washed twice with PBS to remove any residual virus. Medium containing the drug was then added to each well, followed by incubation for 48 h.

At the end of incubation, CCK-8 solution was added, the OD value of each well was measured, and the viral inhibition rate was calculated to evaluate the antiviral efficacy of the compounds in different modes.

#### 4.2.6. Time-of-Addition Assay for Assessing the Effects of AALE and Parthenolide on Different Phases of the RSV Life Cycle

To further determine the effects of AALE and parthenolide on the viral replication cycle, 1500 μg/mL of AALE or 60 μM of parthenolide was added to the virus-infected cells at 2 h before infection and at 0, 2, 4, 6, 8, 10, and 12 h after infection, respectively [37]. The OD values were measured at a wavelength of 450 nm 48 h later, and the viral inhibition rate was calculated.

#### 4.2.7. Analysis of Gene Expression in Hep-2 Cells Using qPCR

According to the FastPure Cell/Tissue Total RNA Isolation Kit Manual (RC101-01, Vazyme), total RNA from Hep-2 cells was extracted. The total RNA concentration extracted was determined by Nano Drop 2000 (Thermo Fisher Science, Waltham, MA, USA). Real-time fluorescence quantitative PCR reactions were performed using a One Step TB Green^®^ PrimeScript™RT-PCR Kit II (RR086A, Takara). Relative gene expression was calculated using the 2^−ΔΔCt^ method and normalized to the endogenous control GAPDH. The sequences of each gene primer are shown in Table 3 [5].

#### 4.2.8. Western Blot Analysis of Protein Expression in Hep-2 Cells

Precooled RIPA lysis buffer (Beyotime, Shanghai, China) was added to the cells and lysed for 20 min on ice. The protein supernatant was collected, and the protein concentration was determined using the BCA Protein Analysis Kit (Beyotime, Shanghai, China). The total protein was electrophoretically separated on 10–12% SDS-PAGE gel and then transferred to a PVDF membrane. After the membrane was sealed in the blocking solution for 2 h, it was incubated overnight with the primary antibody (Abcam) at 4 °C, then washed three times with Tris Buffered Saline with Tween 20 (TBST), and incubated with the corresponding secondary antibody (Abcam) at room temperature for 2 h. Finally, the membrane was cleaned three times with TBST buffer solution. After adding developer to the membrane, the image was exposed and saved [38].

#### 4.2.9. Cell Immunofluorescence Analysis of RSV-F Protein Expression

First, the cells were fixed at room temperature with 4% paraformaldehyde for 15 min. After washing three times with PBS, they were permeated with 0.3% Triton X-100 for 20 min. After washing three times with PBS, they were sealed with a 5% BSA solution for 1 h. Then, the primary antibody against RSV-F protein (1:2000) was added, followed by incubation overnight at 4 °C. After PBST washing, the secondary antibody (Alexa Fluor 488-labeled sheep anti-mouse immunoglobulin) was added, followed by incubation in the dark at room temperature for 1 h (1:2000). Subsequently, DAPI staining solution was added for staining for 5 min, and images were observed and collected using a fluorescence inverted microscope [39].

### 4.3. In Vivo Experiments

#### 4.3.1. Animals and Experimental Design

Female BALB/c mice aged 6–8 weeks, weighing 15–20 g, were selected for the animal experiments and purchased from the Guangdong Provincial Medical Laboratory Animal Center. The mice were given adequate feed and water every day. This experiment was conducted after review and approval by the Institutional Animal Care and Use/Ethics Committee, Shenzhen Center for Disease Control and Prevention. This experiment was carried out at the ABSL-2 laboratory of the Shenzhen Center for Disease Control and Prevention.

As shown in Table 4, the mice were randomly divided into 7 groups using a random number table, with 6 mice in each group: normal control group, virus control group, ribavirin group (50 mg/kg/d) [5], high-dose AALE group (300 mg/kg/d), low-dose AALE group (100 mg/kg/d) group [40], high-dose parthenolide group (15 mg/kg/g) and low-dose parthenolide group (5 mg/kg/g) [41]. After ether anesthesia, 50 μL of RSV solution was slowly instilled into the nasal cavity of mice. After 24 h, the drug group was administered their respective treatment by gavage at a dose of 0.2 mL/20 g of body weight once a day for 5 consecutive days. The normal group and the virus control group were given a similar amount of normal saline. On the fifth day after infection, the mice were sacrificed, weighed, and their lung tissues and serum were collected for subsequent experiments.

#### 4.3.2. Lung Index, Lung Index Inhibition Rate and Hematoxylin–Eosin (HE) Staining Analysis

After the mice were sacrificed by cervical dislocation, the lung tissues were isolated, the lung weight was measured, and the lung index and the inhibition rate of the lung index were calculated. The left lung was fixed in 4% paraformaldehyde fixation solution and then used to prepare paraffin sections of lung tissue, followed by HE staining. The histological changes in the lung were observed under a light microscope, and the images were recorded [42,43].

Lung index = wet weight of lung/body weight of lung × 100%

Lung index inhibition ratio = (lung weight of model group − lung weight of experiment group)/(lung weight of model group − lung weight of blank control group) × 100%

#### 4.3.3. Quantification of RSV Load and Gene Expression of RIG-I/TLR3 Signaling Pathway Components in Mouse Lung Tissue by qPCR

Total RNA was extracted from mouse lung tissues using the FastPure Cell/Tissue Total RNA Isolation Kit (RC101-01, Vazyme) following the manufacturer’s instructions. The concentration and purity of the isolated RNA were determined using a NanoDrop 2000 spectrophotometer (Thermo Fisher Scientific). mRNA expression levels of RSV-F, RIG-I, TLR3, IRF3, IFN-β, and IL-6 were detected using the One Step TB Green^®^ PrimeScript™ RT-PCR Kit II (RR086A, Takara). Primer sequences for each gene are listed in Table 5 [5].

#### 4.3.4. Measurement of IFN-β and IL-6 Levels in Mouse Serum by ELISA

Blood was collected from mice. After standing at room temperature for 1 h, it was centrifuged at 3000 r/min for 15 min to obtain serum. The contents of IFN-β and IL-6 in mouse serum were detected by the method outlined in the enzyme-linked immunosorbent assay kit (E-EL-M0044, Elabscience). The absorbance value of the solution at a wavelength of 450 nm was determined by a microplate reader to quantify the cytokine level.

### 4.4. Statistical Analysis

All data were statistically analyzed using GraphPadPrism9 statistical software, and data normality and variance homogeneity were confirmed before ANOVA. *p*-value < 0.05 was considered significant. For in vitro experiments, *n* = 3 per group; for animal experiments, *n* = 6 per group.

## 5. Conclusions

The present study demonstrated the anti-RSV activity of AALE and parthenolide in both in vitro and in vivo models. Both AALE and parthenolide can inhibit RSV invasion and replication in host cells, activate the immune protective mechanism of host cells, and enhance the defense ability against pathogen invasion. Their mechanism of action is shown to down-regulate the expression of RIG-I and TLR3 signaling pathway-related genes and proteins. In vivo, they exert antiviral activity by inhibiting the proliferation of RSV and reducing lung injury caused by RSV. These results indicate that AALE and parthenolide have therapeutic potential for the treatment of RSV-induced diseases and are promising anti-RSV agents.

## Figures and Tables

**Table 1:** Cytopathic effect (CPE) of RSV at different dilutions.

Dilution of Virus	Number of Holes	Number of CPE Holes	Number of No CPE	Cumulative Number of CPE Holes	Cumulative Number of No CPE Holes	Total	CPE Ratio	CPE Percentage
10^−1^	6	6	0	25	0	25	25/25	100%
10^−2^	6	6	0	19	0	19	19/19	100%
10^−3^	6	6	0	13	0	13	13/13	100%
10^−4^	6	5	1	7	1	8	7/8	87.5%
10^−5^	6	2	4	2	5	7	2/7	28.6%
10^−6^	6	0	6	0	11	11	1/11	0%

**Table 2:** Screening of *Artemisia* extracts and compounds against RSV.

Extract	IC_50_ (μg/mL)	EC_50_ (μg/mL)	SI
AALE	2713	98.21	27.62
ACBE	2331	103.8	22.46
ACTE	3902	143.1	27.27
Compound	IC_50_ (μM)	EC_50_ (μM)	SI
Dihydroartemisinin	63.87	29.87	2.13
Artemether	279.5	94.59	2.95
Artesunate	57.62	-	-
Costunolide	62.12	18.38	3.38
Parthenolide	75.19	9.19	8.18
Dehydrocostuslactone	26.27	-	-
Atractylenolide	25.93	-	-

**Table 3:** Primer sequences of human.

Gene	Primer Sequence (5′-3′)
*RSV-F*	F:AACAGATGTAAGCAGCTCCGTTATC
	R:GATTTTTATTGGATGCTGTACATTT
*RIG-I*	F:TGATTGCCACCTCAGTTGCT
	R:ACTGCTTCGTCCCATGTCTG
*TRAF3*	F:CTGAACAAGGAGGTTACAAGGA
	R:TTGTCCTCCACGGTCTTCAC
*IRF-3*	F:CAAGAGGCTCGTGATGGTC
	R:GGTAGGCCTTGTACTGGTCG
*IL-6*	F:ATGAGGAGACTTGCCTGGTG
	R:CAGCTCTGGCTTGTTCCTC
*IFN-β*	F:TTCAGTGTCAGAAGCTCCTGTGGC
	R:GCCAGGAGGTTCTCAACAATAG
*TLR3*	F:GAAAGGCTAGCAGTCATCCA
	R:CATCGGGTACCTGAGTCAAC
*GAPDH*	F:ACCCAGAAGACTGTGGATGG
	R:ACACATTGGGGGTAGGAACA

**Table 4:** Design and treatment regimen of RSV mouse model.

Groups	The Number of Mice	RSV Infection (Day 0)	Treatment (Daily, Days 1–5)	Dose	Administration
Normal	6	No	Saline	0.2 mL/20 g/d	Oral gavage
Virus	6	Yes	Saline	0.2 mL/20 g/d	Oral gavage
Ribavirin (Positive Control)	6	Yes	Ribavirin	50 mg/kg/d	Oral gavage
AALE-Low dose	6	Yes	AALE	100 mg/kg/d	Oral gavage
AALE-High dose	6	Yes	AALE	300 mg/kg/d	Oral gavage
Parthenolide-Low dose	6	Yes	Parthenolide	5 mg/kg/d	Oral gavage
Parthenolide-High dose	6	Yes	Parthenolide	15 mg/kg/d	Oral gavage

**Table 5:** Primer sequences of mouse.

Gene	Primer Sequence (5′-3′)
*RIG-I*	F:ATCTGTAAACTCTGTGCCGCC R:CCTCGGAGACATCCTTGGCT
*IRF-3*	F:TGTGATGGTCAAGGTTGTTCC R:GTAAGGGAGATAGGCTGGCTGT
*TLR3*	F:TCTCTGGGCTGAAGTGGACAA R:CTTTGCTTAGTAAATGCTCGCTTC
*IL-6*	F:GTTGCCTTCTTGGGACTGATG R:CTCATTTCCACGATTTCCCAGA
*IFN-β*	F:CTGCGTTCCTGCTGTGCTTC R:CGCCCTGTAGGTGAGGTTGAT
*GAPDH*	F:ATGACATCAAGAAGGTGGTG R:CATACCAGGAAATGAGCTTG

## Data Availability

The original contributions presented in this study are included in the article. Further inquiries can be directed to the corresponding authors.

## Abbreviations

The following abbreviations are used in this manuscript:

RSV	Respiratory syncytial virus
RSV-F	Respiratory syncytial virus fusion protein
AALE	*Artemisia argyi* Levl.et Vant extract
ACBE	*Artemisia caruifolia* Buch-Ham extract
ACTE	*Artemisia capillaris* Thunb extract
CCK-8	Cell Counting Kit-8
qRCR	Quantitative polymerase chain reaction
IF	Immunofluorescence
WB	Western blot
RIG-I	Retinoic acid-inducible gene I
TRAF	TNF receptor-associated factor
IFN-β	Interferon-β
TLR-3	Toll-like receptor-3
IL-6	Interleukin-6
HE	Hematoxylin–eosin
CPE	Cytopathic effect
PBS	Phosphate-buffered saline
DMEM	Dulbecco’s modified Eagle medium
DMSO	Dimethyl sulfoxide
BSA	Bovine serum albumin
DAPI	4′,6-diamidino-2-phenylindole
OD	Optical density
PVDF	Poluvinylidene fluoride
RIPA	Radio-immunoprecipitation assay
TBST	Tris-buffered saline with Tween 20
BCA	Bicinchoninic Acid
SI	Selection index
EC50	50% effective concentration
IC50	50% inhibitory concentration
TCID50	Median tissue culture infection dosage
Hep-2	Human laryngeal carcinoma epithelial cells
